# Effectiveness of technology-enhanced teaching and assessment methods of undergraduate preclinical dental skills: a systematic review of randomized controlled clinical trials

**DOI:** 10.1186/s12909-020-02211-4

**Published:** 2020-08-28

**Authors:** Khaled Khalaf, Mohamed El-Kishawi, Shahd Mustafa, Sausan Al Kawas

**Affiliations:** Department of Preventive and Restorative Dentistry, College of Dental Medicine, University of Sharjah, Sharjah, United Arab Emirates

**Keywords:** Effectiveness, Technology-enhanced, Teaching, Assessment, Undergraduate, Dental preclinical skills, RCT, Review

## Abstract

**Background:**

To investigate the effectiveness of technology-enhanced teaching and assessment methods of undergraduate preclinical skills in comparison to conventional methods.

**Methods:**

A comprehensive search strategy was implemented using both manual and electronic search methods, including PubMed, Wiley, ScienceDirect, SCOPUS, and the Cochrane Central Register of Controlled Trials. The search and selection of articles that met the inclusion criteria were carried out in duplicates. A Cochrane data extraction form for RCTs was used to extract the relevant information from all included articles. Risk of bias of all included articles was assessed independently by two authors using the Cochrane risk of bias tool.

**Results:**

A total of 19 randomized controlled clinical trials met the inclusion criteria and were included in this review. The majority of the studies included in this review have a high risk of bias mainly due to incomplete data, lack of blinding of the examiners, and due to other biases, such as small sample sizes, not accounting for additional hours of training, and the lack of calibration of examiners grading the preparations. Conflicting results were reported in the included studies with regards to whether there were differences between the intervention and control groups in the outcome measure of quality of students’ performance. A meta-analysis could not be done for this study due to the heterogeneity among the included studies.

**Conclusions:**

Technology-enhanced teaching and assessment tools used in preclinical skills training of undergraduate dental students have the potential to improve students’ performance. However, due to the conflicting outcomes reported in the 19 studies included in this systematic review and their high risk of bias, better quality studies are required to find a definitive answer to the research question of this systematic review.

## Background

According to the American Dental Association (ADA), producing competent dental graduates is an aim that dental schools thrive to achieve [1]. This can be accomplished through a good curriculum that helps them to continue honing their skills and knowledge over their lifetime and serve their communities [2]. One method of testing the effectiveness of a curriculum is through students’ assessment of the learning outcomes stated in the curriculum. There are two main forms of students’ assessment, formative and summative assessments. Formative assessment evaluates the learning process of students at any point during the teaching program through methods such as self-reflection, in-course assignments, and course-feedback. This improves the quality of learning through understanding of the individual strengths and weaknesses. Also, it aids as a reflection on students’ strategies to improve their performances. Summative assessment, on the other hand, is mainly implemented at the end of a course or program or at strategic stages of a course or a program and may include a variety of assessment methods such as, written exams, Objective Structured Clinical Examination (OSCE), oral and clinical skills exams. While the latter form of assessment can promote motivation through recognition of achievements that the student has obtained, it does not allow for students to reflect on areas that require improvement. It is very important to have a good balance of both forms of assessment, as focusing solely on the summative type may result in lower quality learning, while focusing on the formative type can lead to students not achieving the level of competency required in their course or program [3, 4].

Traditionally, preclinical training in dentistry relied on practicing skills on plastic teeth under the supervision of dental experts [5]. These plastic teeth are typically placed in jaws within a dental simulator. All preparations done on the plastic teeth are then checked and graded by an experienced dental instructor. The use of traditional typodont (manikin-head) has always been considered a valuable tool for simulating patient care procedures [6]. The advantages of using these methods may include low cost, an effective method of improving hand-eye coordination and manual dexterity, and the fact that it has good long-term credibility being the method of choice for decades in preclinical dental training. However, there are also major drawbacks, including the inability to calibrate the evaluation process due to the general focus on task outcome and a heavy reliance on the instructor’s subjective evaluation. This highlights a lack of consistency in students’ evaluations, even when evaluating the same work on different occasions. In order to overcome these limitations, computerized dental teaching and assessment systems were suggested as potential alternative feedback and assessment tools that can improve student’s learning and self-assessment experiences [7].

Recently, there has been an evolution in the development and implementation of computerized technologies such as virtual reality, augmented reality, and haptic technology with feedback in dental training. Virtual reality is a computer-simulated environment [8]. Augmented reality refers to a form of technology that integrates both a real environment and a virtual environment to provide an immersive experience [9]. Haptic technology is a more recent form of technology that involves tactile sensation while interacting with computer-generated objects [10]. All of these forms of technology could potentially enhance the learning and teaching of manipulative skills, particularly during preclinical training [11–14]. However, there are many disadvantages that are associated with technology-enhanced assessment systems. The systems are typically very expensive and require training for both staff and students, particularly if the assessment tool is complex. Due to the fact that this would require considerable funding and resources in order to prepare both staff and students in its use, technology-enhanced assessment tools should be able to enhance learning and teaching of practical skills at a greater level than the traditional method of assessment in order to justify the cost and time. Therefore, the aim of this systematic review was to investigate the effectiveness of technology-enhanced teaching and assessment methods of undergraduate preclinical skills in comparison to conventional methods.

## Methods

### Protocol and registration

This systematic review was conducted according to PRISMA guidelines (Preferred Reporting Items for Systematic Review and Meta-Analysis) [15] and was registered at the Open Science Framework database (https://osf.io) under the registration code: osf.io/xvm7t.

### Eligibility criteria

Studies which fulfil the following criteria were selected:

- *Population:* undergraduate dental student’s preclinical skills
- *Intervention*: technology-enhanced teaching and assessment methods including but not limited to digital scanners, virtual reality, augmented reality, and haptic technology
- *Comparison*: conventional teaching and assessment methods (using a manikin and manual assessment using a periodontal probe, explorer, or/and mouth mirror by a dental instructor)
- *Primary Outcome measure*: effectiveness of technology-enhanced assessment tools when compared to conventional assessment tools in terms of minimizing procedural errors
- *Secondary outcome measures:* student satisfaction, time taken to complete the preparation
- *Study design*: randomized controlled clinical trials
- *Publication dates:* No limit on the date of publication was applied. The search was conducted until January 2020.
- Exclusion criteria: studies that included non-dental students, post-graduate dental students, did not have full-text articles written in English, or were not randomized controlled trials were excluded.

### Information sources

All studies were obtained through a comprehensive search strategy using electronic and manual search methods to locate both indexed and non-indexed articles. The electronic search was performed with the guidance of a formally qualified librarian. Furthermore, hand-searching of reference lists of the included articles were also examined. The electronic search strategy included the following databases: PubMed, Wiley, ScienceDirect, SCOPUS, and the Cochrane Central Register of Controlled Trials.

### Search strategy

The above databases and hand searching were performed independently by two reviewers (SM and ME). Any disagreements between them was resolved by discussion and reaching a consensus, but if they were unable to reach such a consensus a third reviewer was consulted (KK). A search strategy was developed using a combination of MeSH, non-medical terms, and keywords based on the above PICO domains. The following keywords were used to search the databases following advice from a formally qualified librarian and were adapted to each database respectively.

1. dent* AND (student OR assess* OR evaluation) AND preclinical AND (“technology-enhanced” OR virtual OR haptic);
2. “dental student” AND (assess* OR evaluation) AND preclinical AND (“technology-enhanced” OR virtual OR haptic OR simulation);
3. (dental OR dentist) AND (student OR undergraduate) AND preclinical AND (“technology enhanced” OR haptic) AND (assessment OR competency);
4. dent* AND (student OR undergraduate) AND preclinical AND (“technology-enhanced” OR haptic OR simul* OR virtual) AND (assess* OR competency);
5. dent* AND (student OR undergraduate) AND preclinical AND (“technology-enhanced” OR haptic OR digital OR 3D OR simul* OR virtual OR computer OR e-learning).

The manual hand-search included the following four journals:

1. Journal of Dental Education (2000–2019)
2. European Journal of Dental Education (2000–2019)
3. International Journal of Technology Assessment in Healthcare (2001–2019)
4. Medical Teacher (2000–2019)

### Data extraction

A Cochrane data extraction form for Randomized Controlled Trials (RCTs) was used in this systematic review. Data was extracted independently by two reviewers recording the following items: author, year of publication, sample size, study setting, year of study, discipline being assessed, the technology-enhanced assessment intervention, the main findings, the grading assessment method, faculty calibration and the grading rubric. A summary of this information can be seen in Tables 1 and 2. A meta-analysis was not performed because of the great methodological heterogeneity among the studies examined, mainly because of the different technology-enhanced assessment methods, different disciplines involved, different levels of students, as well as different grading assessment methods. The majority of the studies included had a moderate or high risk of bias.

**Table 1:** Summary of the Data from the Studies Included in this Review

Author, Year	Sample size	Setting	Year of study	Discipline	Technology-enhanced assessment method	Main findings
LeBlanc et al., 2003 [16]	68	Columbia University School of Oral and Dental Surgery, United States	Second Year	Operative Dentistry	DentSim Virtual Reality System	There is no significant difference in overall final performance scores between the groups, but the experimental group improved significantly more than the control group.
Quinn et al., 2003 [17]	32	Dublin Dental School, Republic of Ireland	Second Year	Operative Dentistry	Unspecified Virtual Reality Unit	No significant differences between all three groups in cavity quality.
Quinn et al., 2003 [18]	22	Dublin Dental School, Republic of Ireland	Second Year	Operative Dentistry	Unspecified Virtual Reality Unit	There is no significant benefit in using Virtual Reality-based training for preclinical operative training.
Jasinevicius et al., 2004 [19]	28	Case Western Reserve University, United States	First Year	Operative and Prosthodontic Dentistry	DentSim Virtual Reality System	No significant difference in preparation quality or number of preparations made between both the intervention and control groups.
Wierinck et al., 2005 [20]	42	Katholieke Universiteit Leuven, Belgium	First Year	Operative Dentistry	DentSim Virtual Reality System	One experimental group outperformed the control group during the retention test, but overall, the DentSim does not significantly impact manual skill learning in dental students.
Wierinck et al., 2006 [21]	36	Katholieke Universiteit Leuven, Belgium	First Year	Operative Dentistry	DentSim Virtual Reality System	Performance and learning of a cavity preparation task using a simulation unit is not dependent on the frequency of feedback. The simulation system is as effective for training for manual dexterity.
Wierinck et al., 2006 [22]	36	Katholieke Universiteit Leuven, Belgium	First Year	Operative Dentistry	DentSim Virtual Reality System	Presence of VR feedback enhances acquisition and retention of cavity preparation tasks. VR feedback is more beneficial for long-term retention of skill acquisition.
Urbankova, 2010 [23]	79	Columbia University College of Dental Medicine, United States	Second Year	Operative Dentistry	DentSim Virtual Reality System	The experiment group scored significantly higher in the earlier tests, but by the end of the trial, despite the experimental group scoring higher, it was not significant.
Suebnukarn et al., 2011 [24]	32	Not mentioned	Fourth Year	Endodontic Dentistry	Haptic Virtual Reality Simulator training with micro-CT tooth models	No significant difference in error score reduction or task completion time but significant difference in tooth mass removed.
Kikuchi et al., 2013 [25]	45	Tokyo Medical and Dental University, Japan	Fifth Year	Prosthodontics	DentSim Virtual Reality System	The intervention groups had a significantly higher total score when compared to the control group. Preparation time was significantly shorter in the control group.
Gratton et al., 2016 [26]	80	University of Iowa, United States	Second Year	Prosthodontics	E4D Compare software and CEREC prepCheck	There was no significant difference among all groups in regards to technical score and self-evaluation scores.
Tiu et al., 2016 [27]	30	University of Otago, New Zealand	Fourth Year	Prosthodontics	Preppr scanner software	The experimental group outperformed the other groups in overall acceptable preparations
Llena et al., 2017 [28]	43	University of Valencia, Spain	Third Year	Operative Dentistry	Augmentaty Author 1.2 and Augment Viewer software, and Augment app for mobile device	The experimental group had significantly better class I preparations but there was no significant difference in class II preparation quality when compared to control group.
Liu et al., 2018 [29]	66	School of Stomatology of Nanjing Medical University, China	Fourth Year	Prosthodontics	Real-time Dental Training and Evaluation System (RDTES)	The experimental group scored significantly higher compared to the control group.
Nagy et al., 2018 [30]	36	Semmelweis University, Budapest, Hungary	Fourth Year	Operative Dentistry	KaVo Dental Teacher software	The deviations of mean shoulder width, approximal depth, and occlusal width was significantly smaller in the second preparations of the intervention group, while there was no significant difference in deviation between preparations in the control group.
Sadid-Zadeh et al., 2018 [7]	9	University at Buffalo School of Dental Medicine, United States	Second Year	Prosthodontics	E4D Compare software	The E4D Compare software is as effective as conventional faculty supervision in regards to providing instant feedback on full coverage tooth preparations.
Wolgin et al., 2018 [31]	47	Danube Private University, Austria	Third Year	Operative Dentistry	prepCheck (DentsplySirona)	There was no significant difference when using prepCheck and the conventional method of supervision.
Mladenovic et al., 2019 [32]	41	University of Pristina, Serbia	Fourth and Fifth Years	Oral Surgery	Dental Simulator Mobile Application	There was no significant difference in anesthesia success between the two groups, but time to perform anesthesia was significantly higher in the control group.
Murbay et al., 2020 [33]	32	The University of Hong Kong, Hong Kong	Second Year	Operative Dentistry	Moog Simodont Dental trainer (VR-based system)	There was a significant improvement after exposure to the Moog Simodont dental trainer.

**Table 2:** Summary of the Assessment Criteria used in the Studies Included in this Review

Author, Year	Grading assessment method	Faculty calibration for grading	Grading Rubric
LeBlanc et al., 2003 [16]	Two instructors through conventional means	No	Not present
Quinn et al., 2003 [17]	Two independent scorers through conventional means	No	Yes, but not clear
Quinn et al., 2003 [18]	Two instructors through conventional means	No	Yes, but not clear
Jasinevicius et al., 2004 [19]	Two authors through conventional means	Yes	Yes, but not clear
Wierinck et al., 2005 [20]	The DentSim system without feedback mode	N/A	Yes, but not clear
Wierinck et al., 2006 [21]	The DentSim system without feedback mode	N/A	Yes, but not clear
Wierinck et al. 2006 [22]	The DentSim system without feedback mode	N/A	Yes, but not clear
Urbankova, 2010 [23]	Two instructors through conventional means	Yes	Yes, but not clear
Suebnukarn et al., 2011 [24]	One qualified instructor through conventional means	N/A	Yes, a well-defined grading rubric with criteria
Kikuchi et al., 2013 [25]	The DentSim system without feedback mode	N/A	Yes, a well-defined grading rubric with criteria
Gratton et al., 2016 [26]	Three instructors through conventional means and E4D Compare software	Yes	Yes, a well-defined grading rubric with criteria
Tiu et al., 2016 [27]	Preppr scanner software	N/A	Yes, a well-defined grading rubric with criteria
Llena et al., 2017 [28]	One instructor through conventional means	N/A	Yes, a well-defined grading rubric with criteria
Liu et al., 2018 [29]	Two instructors through conventional means	Yes	Yes, a well-defined grading rubric with criteria
Nagy et al., 2018 [30]	The KaVo Dental Teacher software	N/A	Yes, a well-defined grading rubric with criteria
Sadid-Zadeh et al., 2018 [7]	Two instructors through conventional means	Yes	Yes, a well-defined grading rubric with criteria
Wolgin et al., 2018 [31]	Three experienced assessors through conventional means and prepCheck application	Yes	Yes, a well-defined grading rubric with criteria
Mladenovic et al., 2019 [32]	Unclear of how time was measured	N/A	Yes, a well-defined grading rubric with criteria
Murbay et al., 2020 [33]	Three instructors through conventional means and 2 Shape Trios scanner	Yes	Yes, a well-defined grading rubric with criteria

### Risk of bias assessment of individual studies

The quality of included articles was assessed independently by two authors using the Cochrane risk of bias tool (RoB 2.0). An agreement between the two assessors should have been formed prior to reaching a final decision regarding the overall risk of bias of any study. In the case of disagreement, however, a third assessor was consulted to reach the final decision.

The Cochrane risk of bias tool includes seven domains namely: random sequence generation, allocation concealment, blinding of participants and personnel, blinding of outcome assessment, incomplete outcome data, selective reporting, and other types of bias [34]. The overall risk of bias was allocated as follows: an overall grade of low risk was given if all domains were graded as low risk of bias, a grade of unclear risk was given if one or more domains were graded as unclear risk of bias, and a grade of high risk was given if one or more domains were graded as high risk of bias.

### Evaluation of quality of evidence

A quality grade related to the outcome measure was given to the included studies based on the Grading of Recommendation, Assessment, Development, and Evaluation (GRADEpro Guideline Development Tool, gradepro.org). This tool contained 5 domains for rating the quality of evidence as high, moderate, low, or very low [35].

## Results

### Study selection

**Fig. 1:**
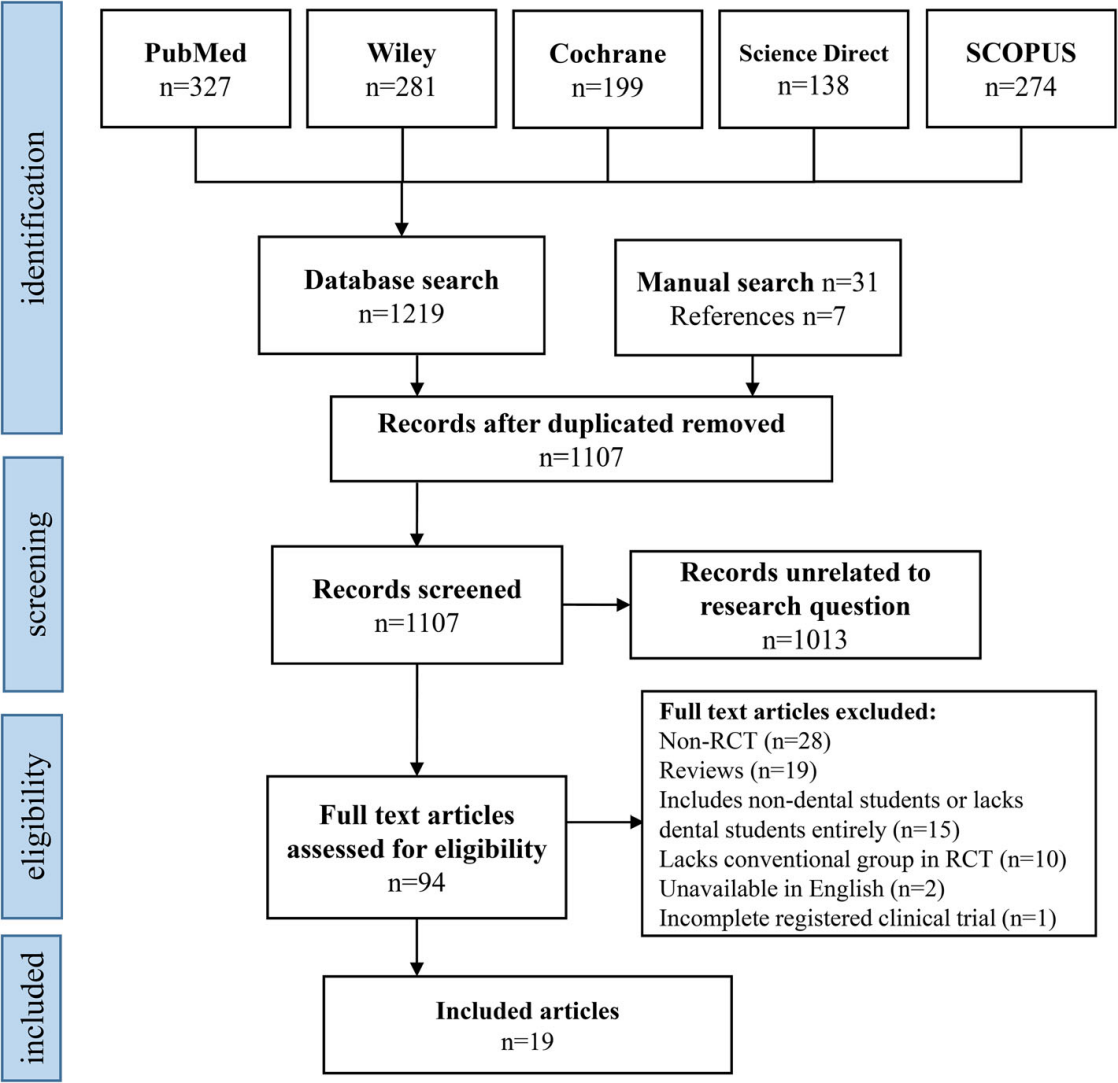
Flow Diagram of Study Identification and Selection using Preferred Reporting Items for Systematic Reviews and Meta-Analyses (PRISMA) format

The kappa statistic for the agreement between the reviewers of searching and selection of studies was 0.86. Following inspection of the titles and abstracts, a total of 1257 articles were initially obtained for assessment, including 1219 from the five electronic databases (PubMed, Wiley, Cochrane Central Register of Controlled Trials, ScienceDirect, and SCOPUS), 31 from the manual hand search of the journals (Journal of Dental Education, European Journal of Dental Education, International Journal of Technology Assessment in Healthcare, and Medical Teacher), and 7 from the reference lists. After removal of duplicates, 1107 articles remained and from those, a further 1013 were removed as they were not directly relevant to the research question of the current systematic review. This left 94 articles for potential inclusion in our systematic review. After reading through the full texts of these 94 articles, 75 were excluded due to various reasons i.e. 28 were not randomized clinical trials, 19 were reviews, 15 did not include dental students entirely, 10 did not include a conventional group for comparison in the randomized clinical trial, 2 were unavailable in English, and 1 was an incomplete registered clinical trial. Thus, a total of 19 studies were included in the final analysis of this review (Fig. 1).

### Study characteristics

Nine studies (47.4%) took place in Europe [17, 18, 20–22, 28, 30–32], five studies (26.3%) took place in North America [7, 16, 19, 23, 26], three studies (15.8%) were conducted in Asia [25, 29, 33], one study (5.2%) was conducted in Oceania [27], and one study (5.2%) did not mention the country setting [24]. One study (5.2%) had a sample size of 9 [7], three studies (15.8%) had a sample size between 11 and 30 [18, 19, 27], 11 studies (57.9%) had a sample size between 31 and 50 [17, 20–22, 24, 25, 28, 30–33], two studies (10.5%) had a sample size between 51 and 70 [16, 29], and two studies (10.5%) had a sample size between 71 and 90 students [23, 26]. Seven studies (36.8%) assessed second year undergraduate dental students [7, 16–18, 23, 26, 33], four (21%) assessed first year [19–22], four (21%) assessed fourth year [24, 27, 29, 30], two (10.5%) assessed third year [28, 31], one (5.2%) assessed fifth year [25], and one (5.2%) assessed a mixture of fourth and fifth year [32]. Most of these studies (57.9%) assessed the student’s ability in operative dentistry [16–18, 20–23, 28, 30, 31, 33]. Five (26.3%) were about prosthodontics [7, 25–27, 29], one (5.2%) was a mixture of both operative dentistry and prosthodontics [19], one (5.2%) about endodontics [24], and one (5.2%) about oral surgery [32]. In total, there were four main types of technology-assessments that were used in all the 19 studies. Ten studies (52.6%) used virtual reality [16–23, 25, 33], six studies (31.5%) used a digital scanner [7, 26, 27, 29–31], two (10.5%) used augmented reality [28, 32], and one (5.2%) used haptic technology [24].

### Risk of bias within studies

**Fig. 2:**
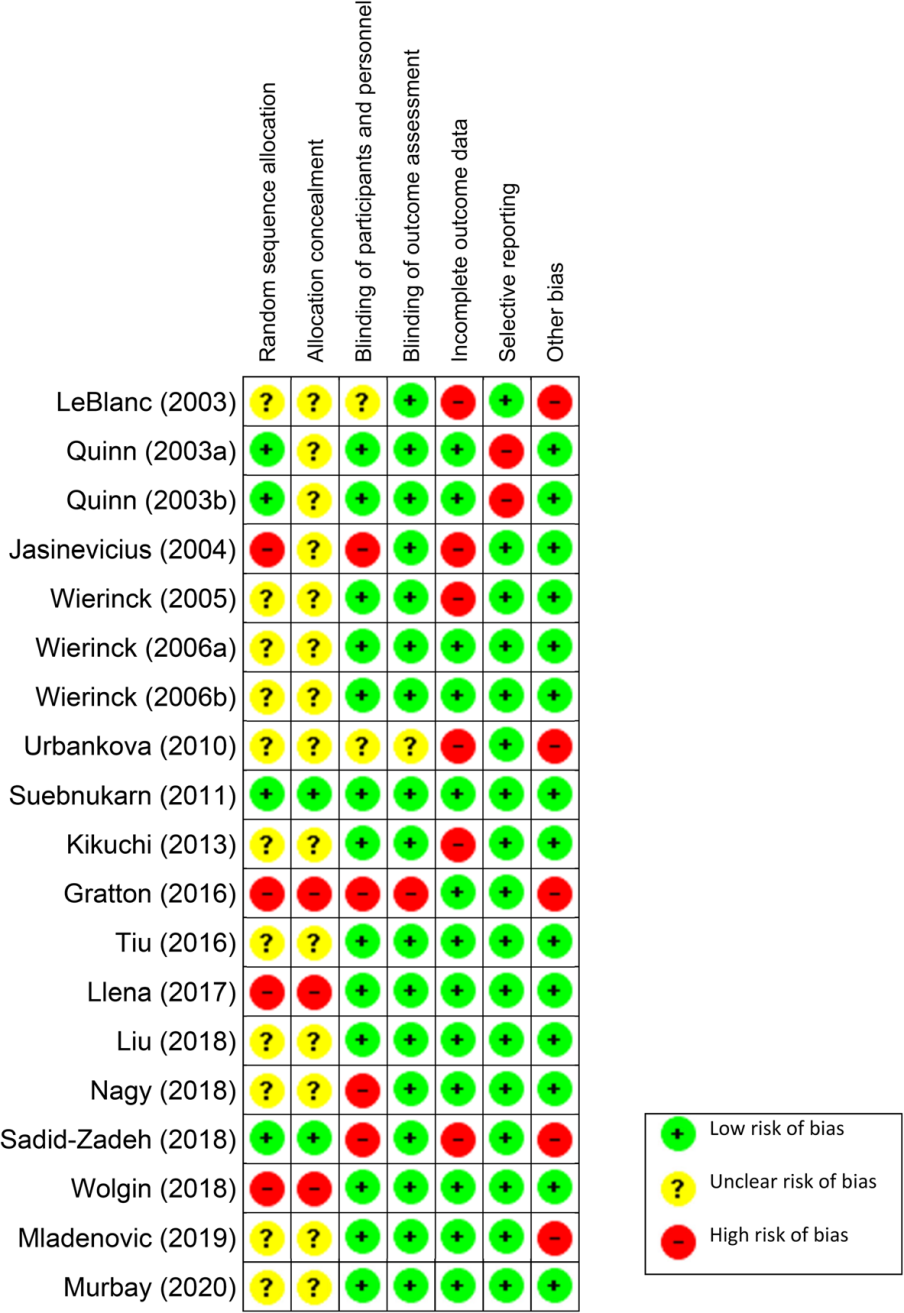
Risk of Bias Assessment for each Included Study

**Fig. 3:**
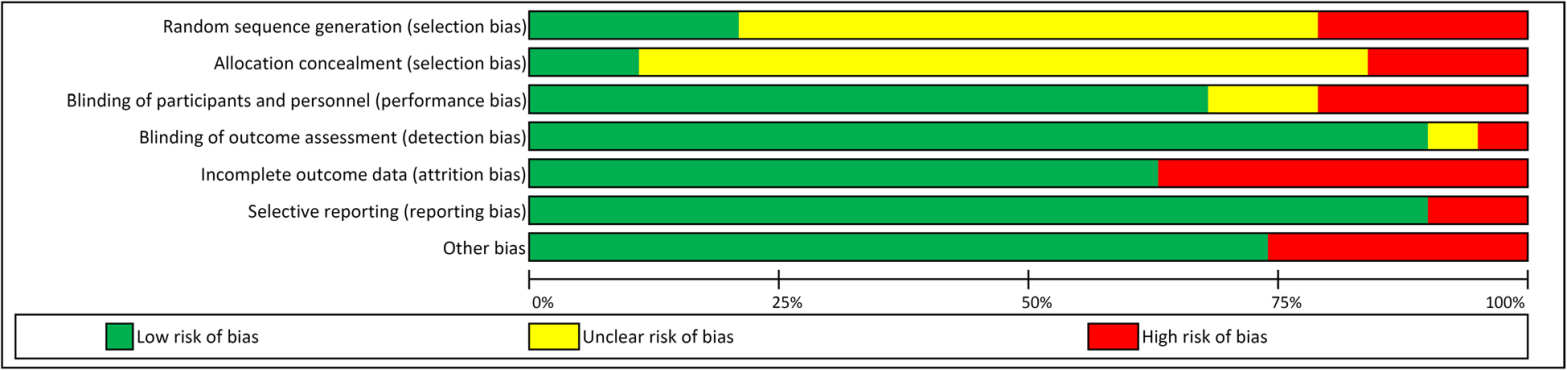
Summary of the Percentage Allocation of Risk of Bias Grades in each Domain Across all Included RCTs

One study had a low risk of bias [24], 5 had an unclear risk of bias [21, 22, 27, 29, 33], and 13 had a high risk of bias [7, 16–20, 23, 25, 26, 28, 30–32], (Fig. 2). A summary of the percentage of allocation of risk of bias grades in each domain can be seen in Fig. 3.

Out of the total 19 studies, 17 had allocation concealment bias [16–23, 25–33], and of these 15 also had another form of selection bias namely, random sequence generation [16, 19–23, 25–33]. Six studies had performance bias in terms of either blinding of the personnel or the participants involved [7, 16, 19, 23, 26, 30]. Six studies had concerns regarding incomplete outcome data [7, 16, 19, 20, 23, 25]. Five studies had other forms of bias such as lack of blinding and calibration of the examiners who are grading the preparations, a small sample size, and a lack of monitoring of additional hours of practice by the students outside of the allocated training time [7, 16, 23, 26, 32]. Two studies had a form of detection bias [23, 26], and two studies had reporting bias [17, 18]. Only one study had no form of bias [24].

### Description of study findings

Five studies provided a questionnaire on student’s experience using a technology-enhanced assessment system, to their intervention group [17, 18, 28, 29, 32]. In two of these studies, most participants believed they could improve their self-learning, self-assessment, and/or assessment abilities using the technology-enhanced method over the conventional method [28, 29]. Two of these studies revealed that most students in the intervention group did not feel that the conventional method would be replaced [17, 18]. In one study, the intervention group reported being more confident in their ability in administering an inferior alveolar dental block in comparison to their peers in the control group [32].

#### Operative dentistry

Eleven studies compared technology-enhanced assessment to conventional assessment in operative dentistry. Two studies by Nagy et al., [30] and Wolgin et al., [31] used a digital scanner to assess cavity preparations in comparison to a control group. Nagy et al., [30] reported that the intervention group had significantly smaller deviations of the mean occlusal width, approximate depth, and shoulder width in their second preparations. In comparison, the control group did not show any significant difference in mean measurements between the first and second preparations. Wolgin et al., [31] reported that using the digital scanner was just as effective as the conventional form of supervision, as there was no significant difference between the intervention and comparison groups in regard to the cavity dimensions.

Five studies specifically used the DentSim Virtual Reality System [16, 20–23]. These five studies all reported an overall increase in performance for the groups involved, however, results of the technical scores of the intervention and control groups varied in these studies. Wierinck et al., [20] reported that the intervention group which used the DentSim without feedback significantly outperformed the control group in performance scores in the retention tests. However, no significant differences were found between the groups in the transfer tests. Another study by Urbankova, [23] found that the intervention group performed significantly better than the control group for the first two examinations, but not on the last examination. A third study by LeBlanc et al., [16] reported that there were no significant differences in overall performance scores between the groups, but that the intervention group improved significantly more than the control group. The last two studies (Wierinck et al., [21]; Wierinck et al., [22]) reported that the intervention groups using DentSim had significantly better performance than the control group in both the immediate and delayed retention tests. The intervention groups, however, took a significantly longer preparation time compared to the control group. In Wierinck et al., [22] study during the delayed retention test and delayed transfer test, only one intervention group differed significantly from the control group, but in the second study by Wierinck et al., [21], both intervention groups had a significantly better performance than the control group in these tests.

Quinn and his co-workers used an unspecified virtual reality machine (Quinn et al., [17] involved two intervention groups and one control group; while Quinn et al., [18] had one intervention and one control group). Both studies reported that generally there were no statistically significant differences between the intervention and control groups. In Quinn et al., [17], the intervention group with real-time and conventional feedback scored significantly higher than the control group in one criteria, the outline form. The rest of the scores in all groups were not statistically significant. The second study by Quinn et al., [18] reported a variation in the significant differences found between the intervention and conventional training groups. Some criteria failed to show significant differences between the two groups, while the remaining criteria showed a significant difference with the virtual reality group showing worse qualitative scores.

Llena’s et al., [28] study used an Augmented Reality Software and mobile application. It reported that there was a significantly higher average score in the intervention group for class I cavity preparations, but no significant differences were observed between the two groups in the class II occlusal box cavity preparation exercise. In another study by Murbay et al., [33], the Moog Simodont was used and reported that there was a significant improvement as a result of being exposed to the dental trainer.

#### Prosthodontics

In prosthodontics, two studies reported the use of the E4D Compare software [7, 26]. E4D Compare scanning software is used as a virtual assessment tool for matching and comparing standard ideal tooth preparation with the operator’s dental work. Sadid-Zadeh et al., [7] reported that the intervention group, interacted only with the software, had consistently a higher percentage of acceptable crown preparations than the control group and the faculty assisted intervention group. All groups showed improvement over time, with undercuts being the most common error, along with unsupported enamel, finish of the preparation, finish line width, amount of occlusal reduction and contour of the preparation. This study found that using the E4D Compare software was just as effective as conventional training. In Gratton et al., [26] study, there was no statistically significant difference between the intervention group and the control group with regards to technical scores and self-evaluation scores during fixed prosthodontics preparation. However, there was a significant difference between the two groups with regards to the average faculty grading, as faculty consistently gave higher average scores than the average E4D Compare grade.

Two studies used another digital scanner as their method of intervention [27, 29]. Tiu et al., [27], reported that the conventional group with no tutor assistance had inconsistent results compared to the intervention group which was able to achieve the acceptable range for preparation finish-line dimensions. By the fourth session, 70% of the intervention group were able to achieve acceptable total occlusal convergence angles (TOC) and finish-line dimensions in their crown preparations. This was outperforming the other groups in the overall acceptable preparations. Liu et al., [29], revealed that there was a significant difference between both the intervention and control groups in practical scores with the intervention group scoring higher than the control group in the overall preparation score.

Kikuchi et al., [25], used the DentSim virtual reality system. This study reported that the intervention groups had significantly higher average scores than the control group. Total scores increased with experience in the intervention groups between experiments, but there was no significant difference in total scores in the control group between experiments. Preparation time was significantly shorter in the control group compared to the intervention groups. The scores for wall incline in the intervention groups were higher than the control group in all experiments. Undercuts decreased with experience, but damage to adjacent teeth was not significantly different among all groups. Scores for margin location in the intervention groups were significantly higher than the control group, but not in chamfer width, wall smoothness, finish line continuity, interproximal clearance resistance, and retention.

The DentSim virtual reality system was also tested in a study by Jasinevicius et al., [19], and was reported to have no significant difference in the number and quality of preparations between both the intervention and control groups.

#### Endodontics

Only one study was related to endodontics by Suebnukarn et al., [24]. A haptic virtual reality simulator intervention was used to evaluate procedural error and treatment time during access cavity preparation. There were no significant differences between the groups with regards to their average error scores, tooth mass removal, and task completion time before training. Error score reduction for both the virtual reality simulator and conventional training groups after training was not significantly different. The intervention group had a significant reduction in tooth mass removal after training when compared to the control group. There was no significant difference in task completion time after training between both groups.

#### Oral surgery

Only one study was related to oral surgery by Mladenovic et al., [32]. It reported that the intervention group had a higher average score and a more limited range of responses on the questionnaire than the control group after using the augmented reality device. The average time for performing anesthesia in the experimental group was significantly lower than the control group. The intervention group had a higher success rate than the control group, but this difference was not statistically significant. Heart rate significantly increased in both groups when performing anesthesia, but there was no significant difference in heart rate between the two groups.

### Assessment of the quality of evidence

The quality of evidence according to GRADE was rated overall as low. Although one RCT was rated as high as it had an adequate sample size and good control of confounding factors and a few limitations, the remaining RCTs were rated as low due to high risk of bias, small sample sizes, conflicting findings and other confounding variables such as, not accounting for additional hours of training, and the lack of calibration of examiners grading the preparations.

## Discussion

This systematic review was designed to assess whether or not there is a difference between technology-enhanced and conventional teaching and assessment methods of preclinical undergraduate dental skills with regards to the quality of the preparation, time taken to complete the preparation and student satisfaction.

From a total of 19 studies that were included in this review, seven reported that there were no significant differences between the intervention and control groups. From these seven studies, two [16, 23] reported that there was a significant improvement rate in their intervention groups. A third study by Quinn et al., [18] had reported that most assessment criteria had no-significant differences, but the criteria that showed significant differences favored the control group.

The remaining 12 studies reported significant differences between the intervention and control groups. Of these, five studies [20, 24, 25, 28, 32] reported that there were significant differences in favor of the intervention groups in some of the exercises or tasks that were not necessarily related to the quality of the preparations or tasks. For example, the time needed to complete the task [25, 32] or only showing significant differences in some of the tasks or exercise criteria that were assigned [20, 24, 28]. This variation in the findings regarding the effectiveness of technology-enhanced teaching and assessment systems may be attributed to the methodology used to assess the students, the type of machine/ system used, and due to the different preclinical courses being assessed.

Formative assessment is an important component of student’s assessment and is usually carried out in preclinical labs through self-assessment or faculty assessment during tooth preparation exercises [4]. However, filling out a self-assessment form does not necessarily improve student ability. In addition, faculty assessment is not considered as an objective method of assessment [36]. The use of digital scanning software, virtual reality, and augmented feedback to visualize students’ preparations in a three-dimensional space may have allowed them to improve the positioning of the handpiece and themselves as they completed their assigned exercises. These technologies also present an objective assessment of measurements of tooth preparations, allowing students to grasp technical skills such as crown reduction, cavity depth, and smoothness at a visual level. Though, if feedback is provided frequently, it can cause a dependency on them, resulting in poor scores during retention practical exams when the feedback option is removed as was seen by Wierinck et al., [21]. However, it should be mentioned that a similar study by the same authors (Wierinck et al., [22]) conflicts with this finding as they found the group with frequently provided feedback performed better. It is not surprising that the effectiveness of student’s feedback in preclinical skills labs may vary greatly between different settings due to several factors. In the conventional method of training, feedback is mainly provided by experienced instructors who are not always present to correct students’ posture and grip on the handpiece during their work. Thus, it is not uncommon for students to wait an extended period of time for faculty feedback. A lower faculty to student ratio which is fairly common in larger preclinical skill labs will contribute to longer waiting times. This can be countered with technology-enhanced assessment systems which offer an instant feedback system, allowing the students to work without waiting for a long period of time. The feedback received from the conventional method of assessment is also subjective, as students may be given different advice on the same preparation from different instructors. Technology-enhanced assessment systems can theoretically reduce the number of instructors needed in a preclinical skills lab and allow a lower faculty to student ratio without sacrificing student performance, as for many systems, a feedback option is available for the student to use. However, more hours may need to be assigned to the staff and students to train them on the use of the system. The cost of supplying an entire preclinical skills lab with technology-enhanced teaching and assessment methods may not be possible for some dental schools.

Summative assessment is another important component of student’s assessment which is typically performed during final examinations [4]. With this in mind, students that have significantly higher hours of practice will have a greater probability of outperforming their peers with a smaller number of hours of practice. Studies by LeBlanc et al., [16] and Urbankova, [23] reported that the intervention group showed a significant improvement compared to the control group despite a lack of significant difference between the two groups in the final score. This may indicate that these systems can promote faster learning in poorly performing students during preclinical lab training. With haptic feedback, students can use tactile sensation to help them differentiate between tooth layers as though they are practicing on a real tooth. The augmented feedback allowed students to view tooth mass loss and handpiece movements during endodontic access preparation, which helped them to control the handpiece better for a more conservative approach.

Although more studies in this systematic review found significant differences between technology-enhanced and conventional teaching and assessment methods, these studies had several limitations and biases. Several studies either did not note or limit the hours that students practiced outside of laboratory working hours or gave the students additional practice hours with the technology-enhanced systems [16, 23, 32]. Blinding of the participants and in some cases the personnel was not possible, and the lack of blinding may have encouraged some students in both groups to work out of hours to outperform or keep up with their peers in the opposing group with regards to the technical score.

There appears to be potential for the use of technology-enhanced teaching and assessment systems in preclinical dental skills to improve the technical and visual experiences, particularly in students who are disadvantaged. However, better quality studies with larger sample sizes are required to find a definitive answer to the effectiveness of these technology-enhanced teaching and assessment systems in preclinical dental skill laboratories. Participants should be randomized using a proper randomization method into an intervention and control group and allocated the same number of hours of practice. If they are allowed to practice outside of laboratory hours, this should ideally be monitored and accounted for. It is preferable that future studies provide both an objective method of assessment using these technology-enhanced systems and a subjective traditional method of assessment using calibrated faculty grading in order to accurately compare the two methods of assessment. Future studies should also focus on using specific technology-enhanced teaching and assessment systems with the same inclusion criteria and measuring similar outcomes such as, quality of the preparation/ procedural errors, time taken to complete the preparation and students’ satisfaction. This would allow us to combine the results and perform a meta-analysis resulting in the provision of a better evidence.

### Limitations of this study

A meta-analysis could not be done for this study due to the significant heterogeneity among the included studies and the high risk of bias found in the majority of the studies.

This systematic review included all forms of technology-enhanced teaching and assessment systems such as virtual systems, augmented reality systems, and digital scanners. Even within the same assessment system, different machines do not necessarily work the same way. This introduces difficulty in applying the findings of one system to another with certain degree of accuracy. Furthermore, different undergraduate dental courses and disciplines were included. As a result, the studies did not necessarily measure the same outcomes in the preclinical courses which made it difficult to accurately compare individual studies. For example, the use of technology-enhanced teaching and assessment systems in endodontic exercises would have different parameters compared to those used in operative dentistry.

The majority of the studies included in this review had a high risk of bias mainly due to incomplete data, lack of blinding of the examiners, and due to other biases, such as small sample size, not accounting for additional hours of training, and the lack of calibration of examiners grading the preparations. Many studies either reported that student training hours were not monitored outside of training time or did not specify that these outside-of-training-time hours were controlled [16, 23, 32]. This may have led to a skew of the result outcomes for many reasons. It is very likely that students who saw their peers using a new method of teaching and grading may have felt that they were behind in regards to clinical skills acquisition and thus may have chosen to stay after-hours in the training laboratories to improve their skills in order to keep up with their peers. On the other hand, students in the groups that use technology-enhanced teaching and assessment systems may also have felt overconfident in their practical skills and in turn chosen to not attend extra training sessions, making the difference between both groups being due to the number of hours practiced rather than the type of teaching and assessment method.

## Conclusions

Technology-enhanced teaching and assessment tools have the potential to improve learning and performance of undergraduate dental students during preclinical skills training. These tools can be used as an adjunct to complement drawbacks of the current traditional teaching and assessment methods. However, further studies with standardized and better design are required to form a definitive answer to the research question posed in this systematic review.

## Data Availability

All data generated or analysed during this study are included in this published article.

## Abbreviations

ADA: American Dental Association
OSCE: Objective Structured Clinical Examination
PRISMA: Preferred Reporting Items for Systematic Review and Meta-Analysis
PICO: Problem/Patient/Population, Intervention/Indicator, Comparison, and Outcomes
RCTs: Randomized Controlled Trials
RoB: Risk of Bias
GRADEpro: Grading of Recommendation, Assessment, Development, and Evaluation
